# Screening Evaporative Dry Eyes Severity Using an Infrared Image

**DOI:** 10.1155/2021/8396503

**Published:** 2021-08-24

**Authors:** Qing Zhang, Yi Wu, Yilin Song, Guanghao Qin, Lanting Yang, Sumeet Singh Talwar, Tiezhu Lin, Gagan Deep Singh Talwar, Hongda Zhang, Ling Xu, Jonathan E Moore, Emmanuel Eric Pazo, Wei He

**Affiliations:** ^1^He Eye Specialist Hospital, No. 128 North Huanghe Street, Shenyang, China; ^2^The Second Affiliated Hospital of Dalian Medical University, Dalian, China; ^3^Tianjin Medical University, Tianjin, China; ^4^Wenzhou Medical University, Jiaxing, China; ^5^Cathedral Eye Clinic, 89–91 Academy Street, Belfast, UK

## Abstract

**Background:**

Dry eye disease (DED) is a multifactorial and one of the most common problems treated in an ophthalmic outpatient clinic. Due to the variability in presentation, diagnosis of DED consists of a combination of subjective and objective clinical tests. The purpose of this study was to assess the effectiveness of a handheld smartphone-based infrared thermal (IRT) camera for screening symptomatic evaporative DED.

**Methods:**

This observational sex-matched control study assessed IRT images of 184 right eyes (46 normal and 138 DED) of 184 participants. Evaporative DED was assessed using noninvasive tear breakup time, fluorescein staining, and the Chinese version of the ocular surface disease index (C-OSDI) questionnaire and categorized into their respective dry eye symptomology group (none, mild, moderate, or severe). The ocular surface temperature (OST) at 8 anatomical regions of interest (ROI) (nasal conjunctiva, nasal limbus, nasal cornea, central cornea, inferior cornea, temporal limbus, temporal cornea, and temporal conjunctiva) were measured and compared using a handheld smartphone-based IRT camera. The effectiveness of these 8 ROIs OST in detecting varying severity of DED was evaluated in terms of correlations with severity of DED and their area under the curve (AUC).

**Results:**

OST at the 8 anatomical ROI was significantly higher in DED participants than in the non-DED group (*p* < 0.05) except for inferior cornea, temporal limbus, and temporal conjunctival regions (>0.05). Analyzing 8 anatomical ROIs revealed that the nasal limbus had the highest Pearson correlation with the severity of DED (0.64, *p* < 0.001). Additionally, the nasal limbus ROI achieved the highest AUC of 0.79 (CI: 0.73–0.85; *p* < 0.05), sensitivity, and specificity (0.96 and 0.91) when comparing its ability to discriminated DED vs. non-DED eyes.

**Conclusions:**

Rather than a diagnostic tool, handheld smartphone-based IRT images can be considered as a rapid, noninvasive, and hygienic screening tool in discriminating DED and non-DED and potentially alleviating inconvenience experienced during conventional tests.

## 1. Introduction

Dry eye disease (DED) is a prevalent ocular condition that may result in ocular pain, decreased visual acuity, and decreased eyesight and quality of life [1–3]. It reported that 25% of patients who come to ophthalmic clinics have symptoms of the dry eye [4, 5]. Due to its multifactorial nature, various internal and external factors can provoke instability of the tear dynamics [6, 7]. Tear film instability is agreed to be one of the critical mechanisms leading to ocular surface inflammation in DED [8, 9]. Evaporative dry eye due to meibomian gland dysfunction (MGD) is the most common type of DED and is primarily caused by poorly functioning or nonfunctioning meibomian glands [10, 11]. Since meibomian glands are modified sebaceous glands located in the upper and lower eyelids, their ducts end along the eyelid margins and release meibum, a lipid component of tears [10]. Tear lipid layer dysfunction may result in increased tear evaporation and clinical presentation of ocular surface illness and inflammation [12]. Diagnosis of DED consists of a combination of objective tests along with a subjective questionnaire [6, 13, 14]. Various objective tests, such as the Schirmer test and tear film breakup time (TBUT) test, and rose bengal are helpful; however, they are clinically semiinvasive and time-consuming, and in mild to moderate dry eye disease, these tests often lack concordance [15]. A sensitive and specific noncontact, rapid, and hygienic screening test for DED could greatly assist clinical utility.

Infrared thermography (IRT) in the medical setting has been used to noninvasively assess the surface temperature of various body parts, organs, tissues, and cells for indication of abnormality [16–19]. It has gained popularity in medical diagnostics and research since the 1960s due to its nonionizing and noninvasive nature [20, 21]. To date, studies have successfully reported the capability of IRT in screening breast cancer, diabetic neuropathy, mass fever screening, and meibomian gland dysfunction [22–25]. Although, recent and past studies have documented the difference in OST profiles between normal and DED eyes [26–31], assessing the severity of DED is of the utmost importance as it usually determines the course of treatment and outcome [32]. However, temperature differences between various ROIs on the ocular surface for assessing the severity of DED have not been explored.

## 2. Methods

### 2.1. Study Design and Participants

This study was conducted in compliance with the tenets of the Declaration of Helsinki and the Institutional Review Board of the He Eye Specialist Hospital, Shenyang, China (IRB-2019K002.0). Random volunteers were recruited from the He Eye Specialist Hospital, Shenyang, China, and informed consent was acquired from all participants after a thorough description of the purpose and implications of the study. All data pertaining to participants in this study were anonymized. After obtaining informed consent, 184 consecutive Asian people (138 with DED eyes and 46 without DED eyes) were participated in an observational, sex and age matched research.  Table 1 contains demographic information.

DED diagnostic was based on the Japanese criteria, as suggested by Uchino, Yuichi, et al. [33]: (1) presence of dry eye symptomatology using Chinese version of ocular surface disease index (C-OSDI) (Allergan Inc., USA); (2) presence of tear film disturbance; and (3) presence of conjunctivocorneal epithelial damage. The presence of all three criteria was needed to establish a positive DED diagnosis.

Exclusion criteria: previous ocular surgery or trauma, age less than 18 years old, chalazion section, acute inflammation, history of blepharal and periorbital skin disease or allergies in 1 month, severe dry eyes with corneal epithelial defect, limbic keratitis, pterygium, corneal neovascularization, glaucoma, breastfeeding, rheumatic immune systemic diseases, history of herpes zoster infection, pregnancy, contact lens wearers, and fluorescein allergy were excluded from the study.

### 2.2. Clinical Evaluation

Noninvasive initial tear film breaking time was assessed using the Keratograph 5M (Oculus, Germany) topographer. Three sequentially readings were captured, and the median value was included in the final analysis. The median value was recorded. Following the methods of Arita et al. [34], conjunctivocorneal epithelial staining assesses corneal and conjunctival epithelium damage (FS score). The double vital staining approach with two microliters of a preservative-free solution containing 1% Lissamine green and 1% sodium fluorescein was instilled in the conjunctival sac. The eye was sectioned into three equal pieces (temporal conjunctiva, cornea, and nasal conjunctiva). Each region received a maximum staining score of three points and a minimum of zero points. The combined scores from all three parts were then recorded on a scale ranging from 0 (normal) to 9 (severe) [35].

### 2.3. Questionnaire

Chinese translated and validated C-OSDI (Allergan Inc, Irvine, USA) version was used to assess and quantify DE symptom. The 12 items of the questionnaire can be tabulated into a score that ranges from 0 (no symptoms) to 100 (severe symptoms) points [36].

### 2.4. Infrared Thermal Camera

**Figure 1:**
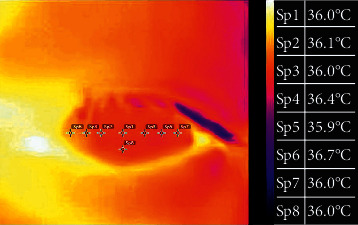
The method in marking the ocular surface and OST acquisition. A typical representation of ocular IRT image as captured by the IRT camera and assessed by FLIR Tools software. The temperature distributions along 8 anatomical regions of interest are recorded instantaneously. Sp1, central cornea; Sp2, nasal cornea; Sp3, temporal cornea; Sp4, nasal limbus; Sp5, temporal limbus; Sp6, nasal conjunctiva; Sp7, temporal conjunctiva; Sp8, inferior cornea.

Participants underwent a noninvasive IRT camera assessment of each right eye (OD) using FLIR One Pro (FLIR Systems Inc., USA) [37] which captured thermographic images of the ocular surface at a resolution of 1440 × 1080 pixels visual resolution (160 × 120 pixels thermal resolution) by a trained ophthalmologist (QZ). The captured images were analyzed using FLIR Tools (version no. 5.13.17110.2003) software designed by FLIR Systems Inc., USA, for personal computer. The OST was measured at 8 anatomical regions of interest (ROI) on the captured IRT images of each OD. A blinded assessor selected the anatomical ROI at the (i) nasal conjunctiva (NConj), (ii) nasal limbus (NL), (iii) nasal cornea (NC), (iv) central cornea (CC), (v) inferior cornea (IC), (vi) temporal limbus (TL), (vii) temporal cornea (TC), and (viii) temporal conjunctiva (TConj) temperature areas, as illustrated in Figure 1.

### 2.5. Measurement Conditions

Before capturing the OST, all participants were required to rest for 10 minutes in the examination room. Participants were asked to blink normally for a few seconds and then to open their OD as wide while the 3 images of the eye were captured immediately in the JPEG format. The IRT images were assessed from 8 AM to 11:30 AM, at a maintained room temperature of 20–23°C and 60–68% humidity. None of the patients included in this study was undergoing any topical or systemic agent for DE or received eye drop installations 6 hours before the capture of thermographic images. None of the patients wear contact lens currently or past. The IRT camera was placed at the slit-lamp support, and the participant's chin was supported on the slit-lamp chin rest to take three consecutive images of the OD always at the same distance. Patients were asked to blink normally for a few seconds and then to open their OD as wide while the 3 images were captured immediately in the JPEG format.

### 2.6. Statistical Analysis

Data are presented as mean ± standard deviation, and all analysis were performed using SPSS version 24 (SPSS Inc., Chicago, IL, USA). The normal distribution of variables was assessed by the Shapiro–Wilk normality test. The analysis of variance test (ANOVA) was utilized to compare OST among different DE severity groups and normal (control) group. Receiver operating characteristic (ROC) curves were used to analyze the screening potential for DED, and the areas under the ROC curves (AUC) were calculated. The average temperature was calculated by assessing the mean value of all the eight ROIs of an individual eye. The level of statistical significance was set at *p* < 0.05.

## 3. Results

In this study, 138 OD with DED (mild: 46 eyes, moderate: 46 eyes, and severe: 46 eyes) and 46 non-DED (control group) OD are given in Table 1. Age and sex among the groups showed no significant differences (*p* > 0.05). NITBUT was significantly shorter (Table 1, *p* < 0.01) in the DED groups in comparison to the non-DED group. Additionally, the C-OSDI score was significantly higher (*p* < 0.001) in the DED groups when compared to the normal group. IRT image assessment revealed that mean nasal region OST was higher than their temporal OST counterpart (Table 2). Furthermore, the highest mean OST was recorded on the nasal limbus anatomical ROI in all groups, while the lowest mean OST was recorded on the temporal conjunctiva anatomical ROI in all groups.

**Figure 2:**
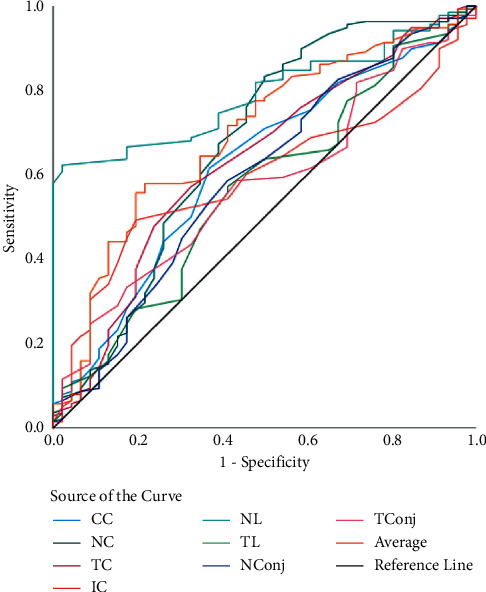
ROC curves of ocular surface temperature for nondry eye disease. ROC curves represent the dry eye disease group (mild dry eye, moderate dry eye, and severe dry eye) vs. the nondry eye group.

**Figure 3:**
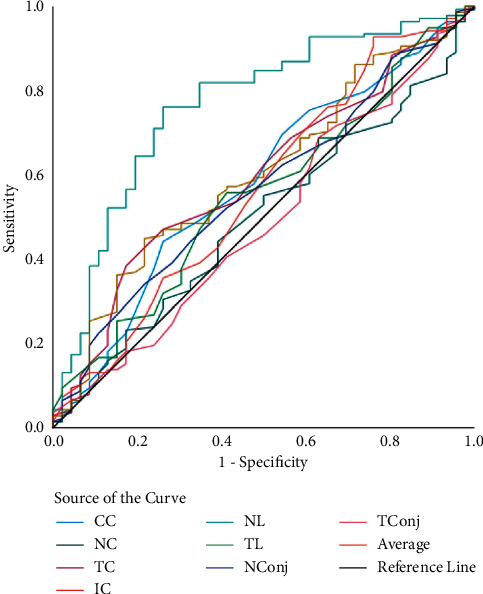
ROC curves of ocular surface temperature for mild dry eye disease. ROC curves represent the mild dry eye disease group vs. nondry eye, moderate dry eye, and severe dry eye groups.

**Figure 4:**
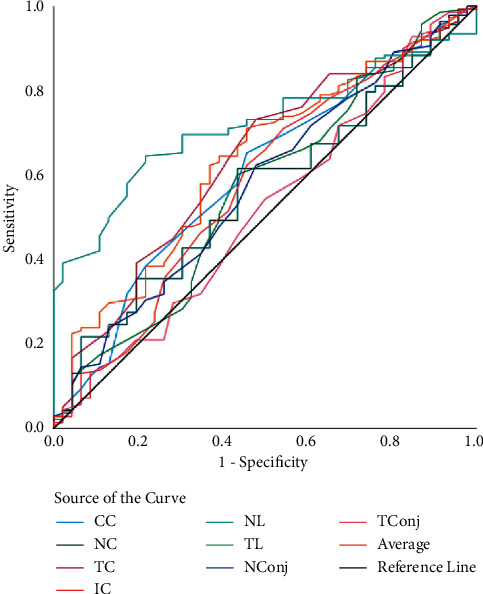
ROC curves of ocular surface temperature for moderate dry eye disease. ROC curves represent the moderate dry eye disease group vs. nondry eye (normal), mild dry eye, and severe dry eye groups.

**Figure 5:**
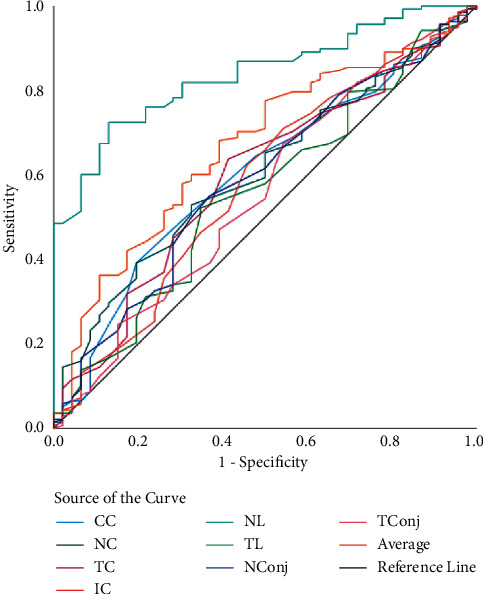
ROC curves of ocular surface temperature for severe dry eye disease. ROC curves represent the severe dry eye disease group vs. nondry eye, mild dry eye, and moderate dry eye groups.

In Table 3, the screening capability for non-DED and DED eyes (model 1) was assessed using 8 anatomical ROI; the ROC curve revealed that the highest AUC of 79% was achieved by NL (Figure 2). The sensitivity and specificity achieved by the nasal limbus region was 96% and 91% (at 34.27°C cutoff; *p* < 0.01). While analyzing mild DED and remaining eyes (model 2), the highest AUC, sensitivity, and specificity was achieved by NL (77%, 94%, and 83%, respectively, at 34.1°C cutoff; *p* < 0.01) (Figure 3). The ROC analysis of moderate DED and remaining eyes (model 3) showed that the highest AUC, sensitivity, and specificity was achieved by NL (72%, 88%, and 85%, respectively, at 36.33°C cutoff; *p* < 0.01) (Figure 4). Finally, ROC analysis of severe DED and remaining eyes (model 4) showed that the highest AUC, sensitivity, and specificity was achieved by NL (84%, 91%, and 70%, respectively, at 36.21°C cutoff; *p* < 0.01) (Figure 5).

Table 4 represents the correlation between various OSTs at different ROIs and DED severity. Two-tailed Pearson correlation revealed that all ROIs except TC and TConj had a nonsignificant correlation. While, the NL region was found to have the highest Pearson correlation (0.63; *p* < 0.01).

## 4. Discussion

This study adds to the body of research that IRT images can be used to measure and discriminate non-DED and DED eyes. Additionally, the findings suggest that the diagnostic ability of handheld smartphone-based IRT images can vary from worthless (DED vs. non-DED; mild DED vs. all; moderate DED vs. all) to good accuracy (severe DED vs. all) using IRT images. The area under ROC curve (AUC) provides a way to measure the accuracy of a test. The accuracy of a test can be classified according to their AUC ratio (excellent = 1–0.9, good = 0.9–0.8, worthless = 0.7–0.8, and not good = 0.6–0.7) [38]. Low levels of accuracy for discriminating the severity of mild and moderate DED using OST is possibly due to the overlapping of signs and symptoms between mild and moderate DED. Screening tools are used in clinical practice to assess the likelihood of a medical condition. The goal of a screening tool is to identify the disease early and possibly leads to a cure or improved survival or quality of life. On the contrary, various screening tests can potentially have an adverse effect and, therefore, one needs to consider when evaluating screening tests, such as their cost, availability, and discomfort [39].

Similar to a previous research conducted on the ocular surface using IRT images, this current research found significantly higher OST at various anatomical ROI in comparison to controls [26, 27, 31, 40, 41]. Furthermore, a different anatomical ROI on the ocular surface has the varying discriminating ability in differentiating DED. The overall NL region was found to be better than other anatomical ROIs in discriminating varying severing of DED. In this current period where sterile environment, hygiene, and distancing are of utmost importance, obtaining a noninvasive assessment of the ocular surface for DED can avoid patient discomfort and maintain hygiene.

Along with other important functions, stable tear film provides lubrication and moisture to maintain good vision and comfort [10]. The outermost lipid layer prevents excessive tear evaporation, lubrication, and microbial protection to the ocular surface [42]. Increased OST variations documented on DED patients [26] and theoretical models [43] of the eye suggest that a stable tear film absorbs while unstable tear film seen in DED allows greater radiation of infrared [30]. Additionally, increased OST can also be attributed to the inflammatory state of the ocular surface in DED conditions [44]. Studies have demonstrated that ocular surface cooling takes place during sustained eye-opening due to the evaporation of tear film, and lack of tears in DED eyes leads to higher and stable OST, even after each blink [30, 41]. Sambursky et al. [45] suggested that 50% of all symptomatic DED patients and nearly all confirmed as DED have significant ongoing ocular surface inflammation. Similar to Kamao et al. [28] and Morgan et al. [46], this current study found that the OST of nasal anatomical ROIs tended to be higher than temporal anatomical ROIs. The nasal anatomical ROIs may be higher due to greater vascularization or possibly related to first tear film breakup in this region [47].

Tests for DED such as tear osmolarity, tear breakup time, Schirmer test, corneal staining, conjunctival staining, and meibomian gland grading have been reported to have sensitivity and specificity of 72.8% and 92%, 84.4% and 45.3%, 79.5% and 50.7%, 54% and 89.3%, 60.3% and 90.7%, and 61.2% and 78.7%, respectively [48]. This finding suggests that acquiring IRT images from anatomical ROI of the ocular surface can result in comparable screening assessment for the severity of DED.

Since AUC is a quantitative representation of overall test accuracy, 50–70% low accuracy, 70–90% represent moderate accuracy, and >90% represent tests with high accuracy [49]. This finding demonstrated that AUC for the NL region for model 1 was 79%, model 2 was 77%, model 3 was 72%, and model 4 was 84%. Since model 3 tested the ability of IRT images to discriminate moderate DED from the cohort (non-DED, moderate DED and severe DED), lower AUC in model 3 could be due to the proximity of OST between mild DED and moderate DED eyes. A meaningful test for DED patients across a broad range of different aetiologies and presentations is a challenge due to a wide range of DED severity [27, 28]. Therefore, it is difficult for a single test result to have adequate sensitivity to function due to its multifactorial nature and several manifestations [50]. Therefore, it is important to keep in mind the overlap between non-DED and DED or even DED severity (mild, moderate, and severe) does exist [51]. The lack of a gold standard test and the absence of concordance is between the signs and symptoms of this disease [52, 53]. Due to this overlap of signs and symptoms in DED between mild and moderate groups, prior studies evaluating DED diagnostic tests such as the tear osmolarity test and lipid layer thickness tests have frequently grouped mild and moderate DED groups [8, 48], thereby improving the discriminatory ability of the tests.

Similar to the findings of other studies [22, 54], this study found that the nasal region of the eye and the average had a better discriminating ability (Table 3). According to the previous body of research, a single point measurement of OST in healthy eyes has been documented to ranges from 34 to 35°C [55]; comparably, this research found that the average mean OST for non-DED participants was 34.70 ± 0.55°C and higher by 0.31°C in the DED participants. Since ocular inflammation is one of the major contributors to DED signs and symptoms [3], it has been postulated to contribute to OST, although the current reports are not comprehensive [44]. As documented by Kim et al. [47], the first tear film breakup tended to occur in different regions of the ocular surface in normal and DED eyes. Therefore, in future studies, it would be interesting to investigate whether the first tear film breakup region influences the OST of the eye.

There are several limitations in this study; findings need to be validated on a larger and diverse ethnic group, including subtypes of DED. Additional IRT instrument used for this study and various other studies assessing the OST has an average accuracy of ±2°C [55]. More accurate IRT instruments in the future would lead to better screening and diagnosis. Severe DED on the far end of the spectrum was excluded from this study as such patients would find it difficult to open their eyes for dry eye assessments as reflex tearing and/or discomfort due to the assessments could falsely alter the OST assessments.

## 5. Conclusion

In summary, the results of this study suggest that IRT images of the ocular surface have comparable results in assessing DED and varying severity of DED. It is rapid, easy to use, and noninvasive, and in this study, it is shown to be a screening tool rather than a diagnostic DED. For diagnosis purposes, IRT images can be used in combination with other conventional tests for assessing the severity of DED.

## Figures and Tables

**Table 1:** Demographic characteristics of participants in the study.

	Non-DED	Mild DED	Moderate DED	Severe DED	*P* value
Number of eyes	46	46	46	46	—
Age (y)	47.22 ± 16.98	46.78 ± 16.72	46.46 ± 16.66	47.17 ± 16.53	0.996
Sex (female%, *n*)	44% (20)	41% (19)	44% (20)	44% (20)	0.376
NITBUT (sec)	11.64 ± 3.53	5.63 ± 2.80	3.54 ± 0.89	2.80 ± 0.76	<0.001
C-OSDI score	9.80 ± 2.01	15.90 ± 4.31	25.23 ± 4.22	36.91 ± 5.17	<0.001

DED, dry eye disease; NIBUT, noninvasive tear breakup time; C-OSDI, Chinese ocular surface disease index.

**Table 2:** Mean ocular surface temperature (OST) variation at various ROI.

ROI	All DED	Non-DED	Mild DED	Moderate DED	Severe DED	*F* value	*P* value
CC (°C)	35.01 ± 0.70	34.76 ± 0.64	34.83 ± 0.70	35.10 ± 0.71	35.09 ± 0.67	3.07	0.03
NC (°C)	35.06 ± 0.65	34.62 ± 0.83	34.98 ± 0.66	35.06 ± 0.66	35.13 ± 0.64	4.77	<0.01
TC (°C)	34.85 ± 0.61	34.58 ± 0.69	34.65 ± 0.59	34.98 ± 0.62	34.92 ± 0.60	4.56	<0.01
IC (°C)	34.83 ± 0.83	34.61 ± 0.57	34.62 ± 0.83	34.92 ± 0.83	34.93 ± 0.80	2.54	0.06
NL (°C)	35.60 ± 0.74	34.93 ± 0.48	34.95 ± 0.70	35.80 ± 0.45	36.05 ± 0.54	6.77	<0.01
TL (°C)	35.57 ± 0.79	35.42 ± 0.80	35.44 ± 0.77	35.64 ± 0.82	35.63 ± 0.77	1.09	0.35
NConj (°C)	34.63 ± 0.72	34.36 ± 0.86	34.45 ± 0.71	34.72 ± 0.74	34.73 ± 0.70	2.84	0.04
TConj (°C)	34.49 ± 0.84	34.32 ± 0.71	34.44 ± 0.83	34.49 ± 0.86	34.54 ± 0.85	0.64	0.59
Average (°C)	35.01 ± 0.60	34.70 ± 0.55	34.81 ± 0.59	35.09 ± 0.57	35.13 ± 0.54	6.32	<0.01

ROI, region of interest; DED, dry eye disease; °C, degree Celsius; CC, central cornea; NC, nasal cornea; TC, temporal cornea; NL, nasal limbus; TL, temporal limbus; NConj, nasal conjunctiva; TConj, temporal conjunctiva; IC, inferior cornea.

**Table 3:** Test effectiveness of ocular surface temperature (OST) at various anatomical ROIs (AUC, sensitivity, specificity, and the selected cutoff values).

Test result variable (s)	Area	Std. error	Significance	Cutoff value (°C)	Sensitivity	Specificity	95% CI
Area under the curve in model 1 (non-DED vs. mild DED, moderate DED, and severe DED)							
CC (°C)	0.62	0.05	0.02	34.25	0.90	0.85	0.53–0.71
NC (°C)	0.67	0.05	<0.01	34.00	0.96	0.76	0.57–0.77
TC (°C)	0.63	0.05	0.01	33.95	0.95	0.89	0.54–0.73
IC (°C)	0.60	0.04	0.05	34.05	0.86	0.91	0.51–0.68
NL (°C)	0.79	0.03	<0.01	34.27	0.96	0.91	0.73–0.85
TL (°C)	0.57	0.05	0.18	34.85	0.86	0.80	0.47–0.66
NConj (°C)	0.60	0.05	0.05	34.05	0.78	0.63	0.50–0.69
TConj (°C)	0.58	0.05	0.12	33.65	0.90	0.83	0.49–0.67
Average (°C)	0.70	0.04	<0.001	34.47	0.89	0.74	0.61–0.78

Area under the curve in model 2 (mild DED vs. non-DED, moderate DED, and severe DED)							
CC (°C)	0.58	0.05	0.09	34.25	0.89	0.87	0.49–0.68
NC (°C)	0.50	0.05	0.94	34.43	0.81	0.85	0.40–0.59
TC (°C)	0.60	0.05	0.05	34.25	0.88	0.80	0.51–0.69
IC (°C)	0.57	0.05	0.18	34.15	0.85	0.74	0.47–0.67
NL (°C)	0.77	0.04	<0.01	34.61	0.94	0.83	0.68–0.85
TL (°C)	0.55	0.05	0.28	34.75	0.89	0.85	0.46–0.65
NConj (°C)	0.57	0.05	0.17	33.95	0.80	0.76	0.48–0.66
TConj (°C)	0.49	0.05	0.89	33.65	0.88	0.89	0.40–0.59
Average (°C)	0.60	0.05	0.04	34.47	0.88	0.76	0.51–0.69

Area under the curve in model 3 (moderate DED vs. non-DED, mild DED, and severe DED)							
CC (°C)	0.60	0.05	0.04	35.35	0.81	0.72	0.51–0.70
NC (°C)	0.56	0.05	0.20	35.48	0.80	0.76	0.47–0.66
TC (°C)	0.63	0.05	0.01	35.30	0.88	0.83	0.54–0.73
IC (°C)	0.58	0.05	0.11	35.65	0.88	0.83	0.48–0.68
NL (°C)	0.72	0.04	<0.01	36.33	0.88	0.85	0.65–0.79
TL (°C)	0.56	0.05	0.20	36.15	0.79	0.72	0.46–0.66
NConj (°C)	0.58	0.05	0.13	35.35	0.89	0.80	0.48–0.67
TConj (°C)	0.52	0.05	0.74	35.38	0.88	0.83	0.42–0.62
Average (°C)	0.63	0.05	0.01	35.42	0.88	0.83	0.54–0.72

Area under the curve in model 4 (severe DED vs. non-DED, mild DED and moderate DED)							
CC (°C)	0.60	0.05	0.04	35.55	0.86	0.80	0.51–0.69
NC (°C)	0.61	0.05	0.03	35.43	0.80	0.74	0.52–0.70
TC (°C)	0.60	0.05	0.05	35.25	0.83	0.78	0.50–0.69
IC (°C)	0.58	0.05	0.09	35.35	0.83	0.74	0.49–0.68
NL (°C)	0.84	0.03	<0.01	36.21	0.91	0.70	0.78–0.90
TL (°C)	0.56	0.05	0.25	36.15	0.80	0.70	0.46–0.65
NConj (°C)	0.59	0.05	0.07	35.15	0.83	0.74	0.50–0.68
TConj (°C)	0.55	0.05	0.28	35.15	0.84	0.76	0.46–0.65
Average (°C)	0.67	0.05	<0.01	35.37	0.87	0.78	0.58–0.76

DED, dry eye disease; °C, degree Celsius; ROI, region of interest; AUC, area under the curve; CI, confidence interval; NC, nasal cornea; CC, central cornea; TC, temporal cornea; IC, inferior cornea; NL, nasal limbus; TL, temporal limbus; NConj, nasal conjunctiva; TConj, temporal conjunctiva.

**Table 4:** Correlation between ROI and DED severity.

ROI	Pearson correlation	Sig. (2-tailed)
CC	0.20	0.01
NC	0.25	<0.01
TC	0.24	<0.01
IC	0.18	0.01
NL	0.63	<0.01
TL	0.12	0.11
NConj	0.20	0.01
TConj	0.01	0.18
Average	0.30	<0.01

DED, dry eye disease; °C, degree Celsius; ROI, region of interest; AUC, area under the curve; CI, confidence interval; NC, nasal cornea; CC, central cornea; TC, temporal cornea; IC, inferior cornea; NL, nasal limbus; TL, temporal limbus; NConj, nasal conjunctiva; TConj, temporal conjunctiva.

## Data Availability

The data used to support the findings of this study are available from the corresponding author upon request.
